# Supplementary material for: 3D U-Net Improves Automatic Brain Extraction for Isotropic Rat Brain Magnetic Resonance Imaging Data
Source: Front Neurosci. 2021 Dec 16;15:801008. doi: 10.3389/fnins.2021.801008 (PMC8716693; doi:10.3389/fnins.2021.801008)
Supplement: Supplementary file 1 [file Data_Sheet_1.PDF]

Supporting information:

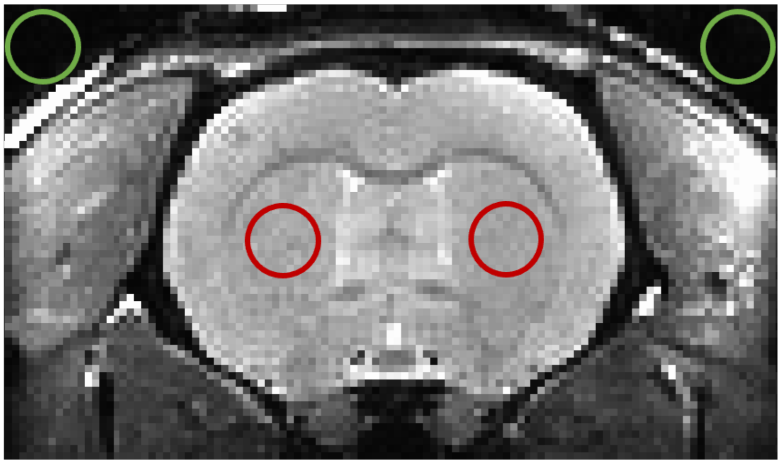

Figure S1. SNR calculation. The SNR was estimated to represent the image noise levels by calculating the ratio of the signal intensity in the area of interest (red circle) to that of the background (green circle).

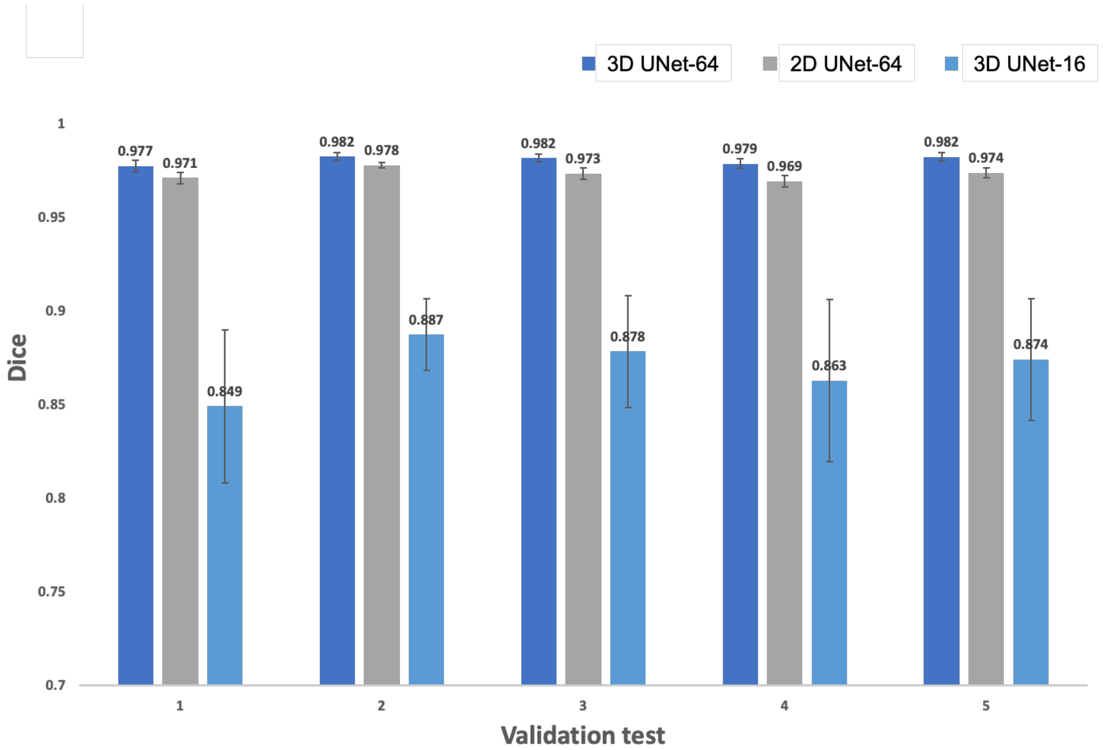

Figure S2. Performance validation results within the training process. In the training process, we randomly selected 80% of the rat data (55 rats) from the training dataset. The remaining 20% of the rat data (14 rats) from the training dataset was used for validating the U-Net model. We repeated this training-validation process five times to avoid randomness bias in data splitting. The U-Net model with highest averaged validation accuracy was then used as the final model for testing.

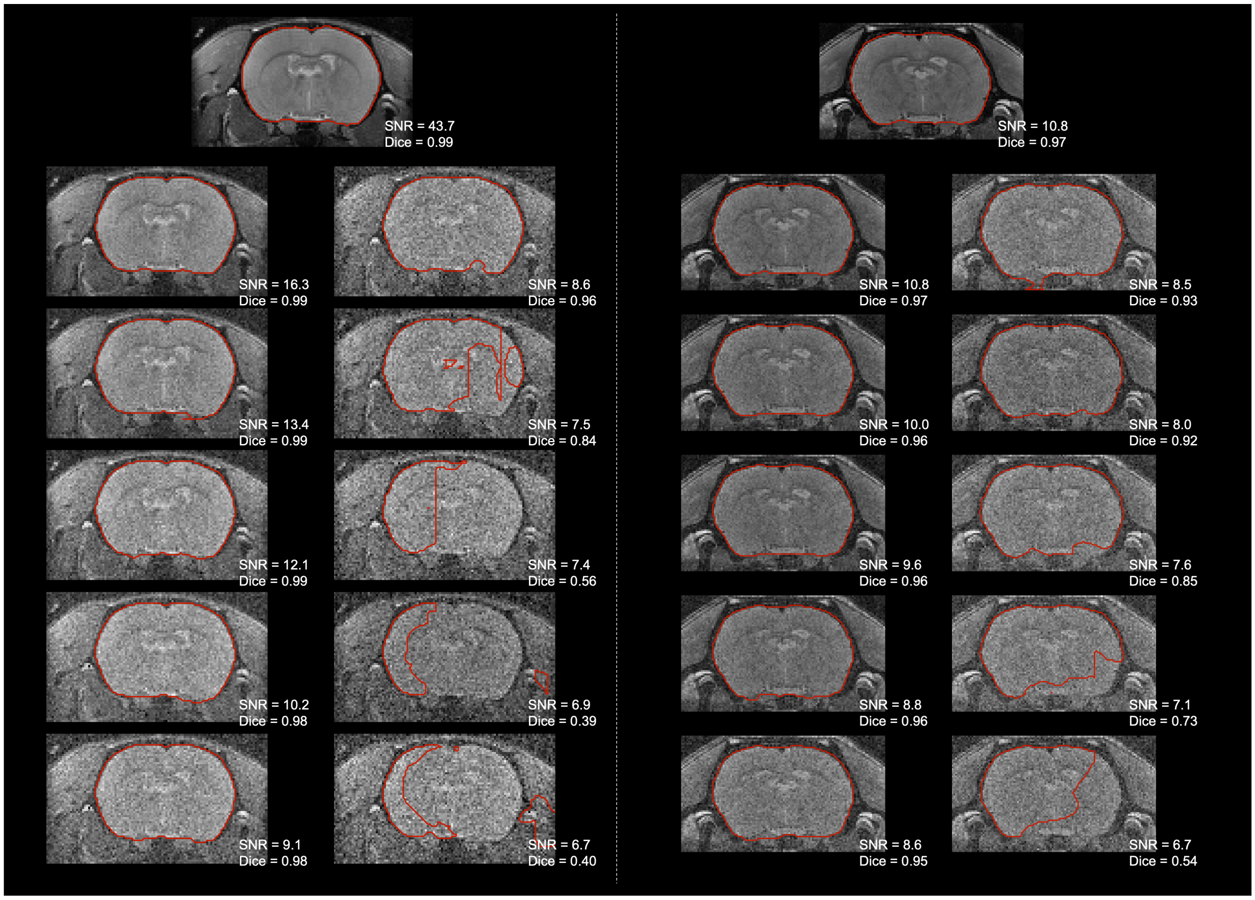

Figure S3. Highest (left panel) and lowest (right panel) Dice for T2w RARE images. For each rat, we added noise with random Gaussian distribution in the normalized testing images with variance from 5×10^{-5} to 5×10^{-4} in increments of 5×10^{-5}. The SNR and segmentation accuracy (Dice) are labeled below each image.

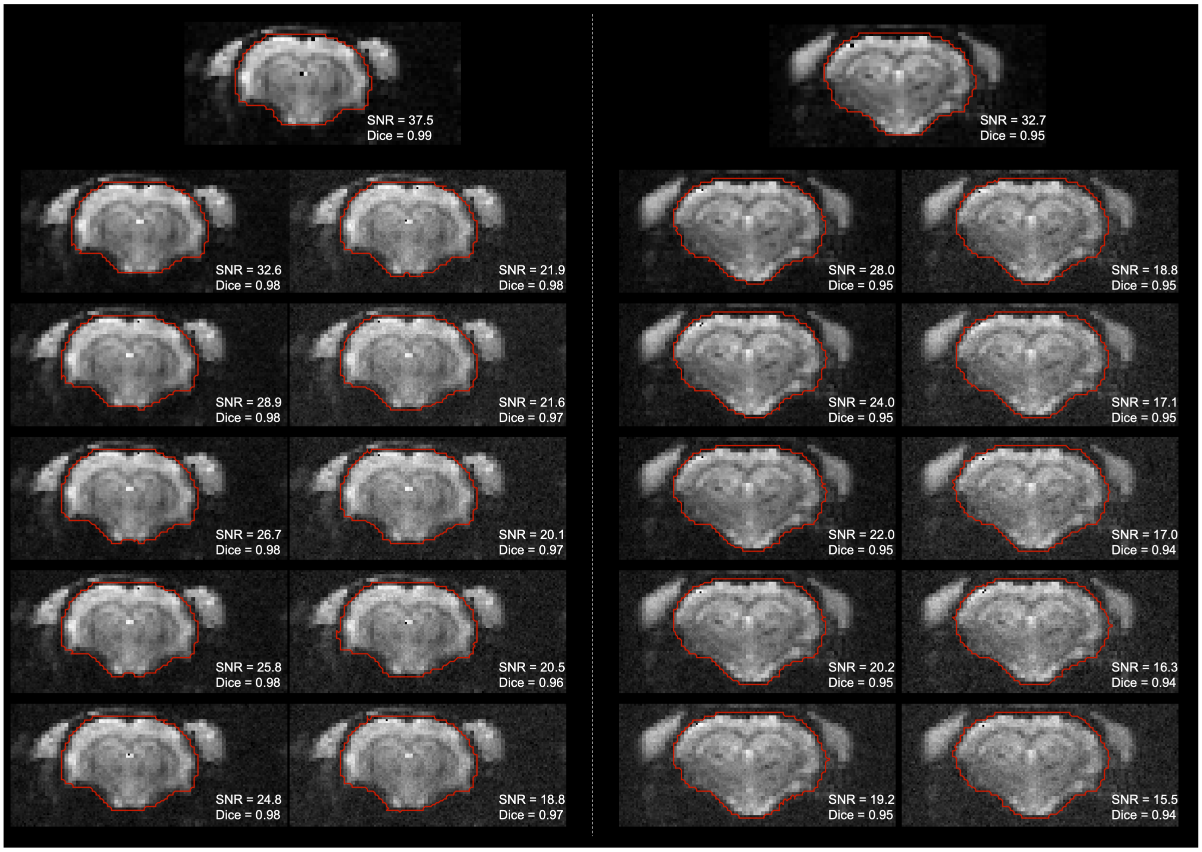

Figure S4. Highest (left panel) and lowest (right panel) Dice for T2*w EPI images. For each rat, we added noise with random Gaussian distribution in the normalized testing images with variance from 5×10^{-5} to 5×10^{-4} in increments of 5×10^{-5}. The SNR and segmentation accuracy (Dice) are labeled below each image.
